# Supplementary material for: Identification of photoperiod-induced specific miRNAs in the adrenal glands of Sunite sheep (Ovis aries)
Source: Front Vet Sci. 2022 Jul 22;9:888207. doi: 10.3389/fvets.2022.888207 (PMC9354845; doi:10.3389/fvets.2022.888207)
Supplement: Supplementary file 1 [file Data_Sheet_1.ZIP › Supplementary file/miRDeep2 file.zip!/miRDeep2 file/Know_Structure/SPLP42A2/oar-mir-374b.pdf]

miRBase precursor : oar-mir-374b
Total read count : 29116
oar-miR-374b read count : 28567
remaining reads : 549

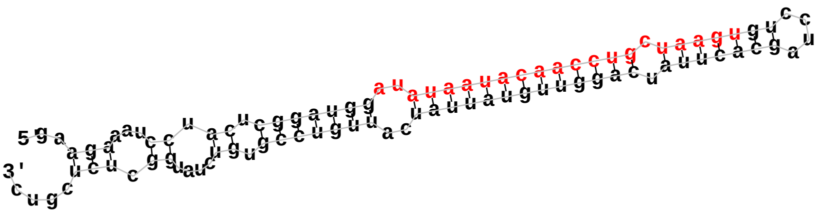

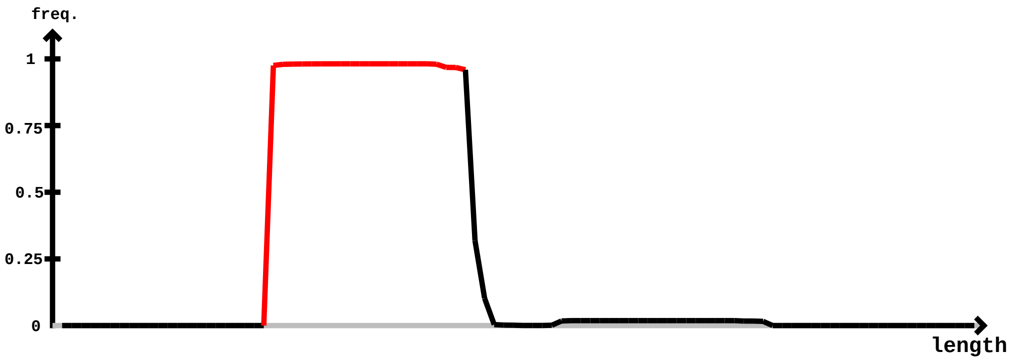


```
                                          oar-miR-374b
5'- gaagaaauccuacucggauggauauaauacaaccugcuaaguguccuagcacuuaucagguuguauuaucauuguccgugucuauggcucucguc  -3'   exp
    ((((..((...((.(((((((..(((((((((((((.(((((((....))))))).)))))))))))))..))))))).))..))..)).))...     reads   mm   sample
    .....................aCauaauacaaccugcu.........................................................     2       1    seq
    .....................auauaauacaaccugcu.........................................................     37      0    seq
    .....................auauaauacaaccugcA.........................................................     1       1    seq
    .....................Cuauaauacaaccugcu.........................................................     6       1    seq
    .....................auauaauacaaccugcua........................................................     342     0    seq
    .....................auauaauacaaccugcuaa.......................................................     10      0    seq
    .....................auauaauacaaccugcuaag......................................................     252     0    seq
    .....................auauaauacaaccugcuaagu.....................................................     18628   0    seq
    .....................auauaauacaaccugcuaagug....................................................     6287    0    seq
    .....................auauaauacaaccugcuaagugu...................................................     2784    0    seq
    .....................auauaauacaaccugcuaaguguc..................................................     41      0    seq
    .....................auauaauacaaccugcuaagugucc.................................................     6       0    seq
    .....................auauaauacaaccugcuaaguguccu................................................     4       0    seq
    .....................auauaauacaaccugcuaaguguccuagcacu..........................................     1       0    seq
    ......................uauaauacaaccugcua........................................................     1       0    seq
    ......................uauaauacaaccugcuaagu.....................................................     23      0    seq
    ......................uauaauacaaccugcuaagug....................................................     7       0    seq
    ......................uauaauacaaccugcuaagugu...................................................     78      0    seq
    ......................uauaauacaaccugcuaaguguc..................................................     7       0    seq
    ......................uauaauacaaccugcuaagugucc.................................................     1       0    seq
    ......................uauaauacaaccugcuaaguguccu................................................     4       0    seq
    .......................auaauacaaccugcuaagu.....................................................     9       0    seq
    .......................auaauacaaccugcuaagug....................................................     2       0    seq
    .......................auaauacaaccugcuaagugu...................................................     3       0    seq
    .......................auaauacaaccugcuaaguguc..................................................     4       0    seq
    .......................auaauacaaccugcuaagugucc.................................................     2       0    seq
    .......................auaauacaaccugcuaaguguccu................................................     3       0    seq
    ........................uaauacaaccugcuaaguguc..................................................     1       0    seq
    ........................uaauacaaccugcuaagugucc.................................................     5       0    seq
    ........................uaauacaaccugcuaaguguccu................................................     10      0    seq
    ........................uaauacaaccugcuaaguguccua...............................................     1       0    seq
    ........................uaauacaaccugcuaaguguccuag..............................................     1       0    seq
    .........................aauacaaccugcuaaguguccu................................................     6       0    seq
    ..........................auacaaccugcuaaguguccu................................................     1       0    seq
    ..........................auacaaccugcuaaguguccuag..............................................     1       0    seq
```

oar-miR-374b

gaagaaauccuacucggauggauauaauacaaccugcuaaguguccuagcacuuaucagguuguauuaucauuguccgugucuauggcucucguc			
..........................auacaaccugcuaaguguccuagc...	3	0	seq
............................acaaccugcuaaguguccuagc...	2	0	seq
....cacuuaucagguuguauuauc.........................	3	0	seq
....acuuaucagguuguauuauc.........................	18	0	seq
....Ccuuaucagguuguauuauc.........................	3	1	seq
....acuuaucagguuguauuaucau.......................	8	0	seq
....acuuaucagguuguauuaucauu......................	1	0	seq
....cuuaucagguuguauuau..........................	1	0	seq
....cuuaucagguuguauuauA.........................	2	1	seq
....cuuaucagguuguauuauc.........................	15	0	seq
....cuuaucagguuguauuauca........................	3	0	seq
....cuuaucagguuguauuaucU........................	1	1	seq
....cuuaucagguuguauuaucau.......................	21	0	seq
....cuuaucagguuguauuaucauu......................	421	0	seq
....uuaucagguuguauuauc.........................	2	0	seq
....uuaucagguuguauuaucau.......................	3	0	seq
....uuaucagguuguauuaucauu......................	36	0	seq
....uuaucagguuguauuaucauugu....................	1	0	seq
....uaucagguuguauuaucauu......................	1	0	seq
